# Comparison of Age-Related Pigmentary Changes in the Auditory and Vestibular Systems Within Mouse and Human Temporal Bones

**DOI:** 10.3389/fnins.2021.680994

**Published:** 2021-05-14

**Authors:** Nicholas S. Andresen, Sarah Coreas, Dillan F. Villavisanis, Amanda M. Lauer

**Affiliations:** ^1^Department of Otolaryngology – Head & Neck Surgery, Johns Hopkins University School of Medicine, Baltimore, MD, United States; ^2^Icahn School of Medicine at Mount Sinai, New York, NY, United States; ^3^Department of Neuroscience, Johns Hopkins University School of Medicine, Baltimore, MD, United States

**Keywords:** melanin, pigmentation, cochlea, vestibular, auditory, age-related hearing loss

## Abstract

**Background:**

Melanin pigmentation is present within the auditory and vestibular systems of the mammalian inner ear and may play a role in maintaining auditory and vestibular function. Melanocytes within the stria vascularis (SV) are necessary for the generation of the endocochlear potential (EP) and decreased EP has been linked to age-related hearing loss. Melanocytes and pigment-containing “dark cells” are present within the vestibular system, but have a less well-defined role. African–American individuals have increased pigmentation within the SV and vestibular system, which is hypothesized to be related to lower rates of age-related hearing loss and vestibular dysfunction. It remains unclear if increased pigmentation confers lifelong protection against hearing loss and vestibular dysfunction.

**Methods:**

Mouse temporal bones were collected from juvenile (3–4 week) and aged (20–32 months) CBA/CaJ mice. Pediatric and adult human temporal bones from Caucasian or African–American individuals were examined from the Johns Hopkins Temporal Bone Collection. Information regarding Fitzpatrick skin type were unavailable, and self-identified race/ethnicity was used as a proxy. Images were taken using light microscopy at 20× magnification. ImageJ software (v1.53) was used to measure pigment within the SV and vestibular system.

**Results:**

In mouse temporal bones pigmentation within the SV increased with age, but pigmentation within the vestibular system did not increase with age. In human temporal bones pigmentation within the SV increased with age and pigmentation within the vestibular system increased within the wall of the utricle, but not other regions of the vestibular system. African–American individuals had higher amounts of pigment within the SV and vestibular system, among both pediatric and adult populations.

**Conclusion:**

Stria vascularis pigmentation increases with age in mouse and human temporal bones. Pigmentation within the vestibular system did not increase with age in mouse specimens and only increased within the utricular wall with age in human specimens. Individuals who identified as African–American had higher pigment content within the SV and vestibular system, both as children and as adults. These results highlight how similar age-related pigmentary changes occur in the auditory and vestibular systems across species and underscore the importance of racial/ethnic diversity in human temporal bone studies.

## Introduction

Pigmentation is present within the auditory and vestibular systems of the mammalian ear. Alfonso Corti first noted the presence of inner ear pigmentation in 1851 (Corti, 1851), however, since this time little has been learned about its function. Pigmentary disorders are associated with inner ear dysfunction (Lezirovitz et al., 2006; Léger et al., 2012; Milunsky, 2017). In humans Waardenburg syndrome and Tietz syndrome cause congenital deafness and hypopigmentation of the hair, eyes, and skin (Milunsky, 2017). Pigmentary disorders have similarly been associated with congenital deafness in dogs and cats (Strain, 2004, 2017). Conversely, differential levels of pigmentation have been hypothesized to alter susceptibility to noise-induced hearing loss and presbycusis (Bunch and Raiford, 1930; Ardiç et al., 1998; Ishii and Talbott, 1998; Murillo-Cuesta et al., 2010; Lin et al., 2011, 2012; Sun et al., 2014), and potentially vestibular dysfunction (Erbele et al., 2016).

Within the cochlea, the primary pigment melanin is produced by intermediate cells (ICs) within the stria vascularis (SV). ICs are melanocyte-like, neural crest progenitor cells that are necessary for the development of the SV, production of endolymph, and development of the endocochlear potential (EP) (Steel and Barkway, 1989; Kim et al., 2013). Precise toxigenic ablation of ICs results in deafness (Kim et al., 2013). In albino mice, the absence of strial melanin is associated with increased age-related EP decline and hearing loss (Ohlemiller et al., 2009). Increased cochlear pigmentation has been observed in African–American individuals (Sun et al., 2014), which correlates with lower risk of noise-induced hearing loss and presbycusis observed in population based studies (Ishii and Talbott, 1998; Lin et al., 2012). Interestingly, in a mouse model of age-related hearing loss, pigment within the SV was observed to increase with age (Kobrina et al., 2020). Similar qualitative observations have been reported in other species (Takahashi, 1971; Keithley et al., 1992; Ohlemiller et al., 2006), raising the question of whether differences in cochlear pigmentation are simply differential responses to the aging process or whether pigmentation has a protective effect.

Less is known regarding the role of melanin in the vestibular system. Previous studies have identified melanocytes within the dark cell areas of the vestibular system (Kimura, 1969; Masuda et al., 1994, 1995). Specifically, melanin has been found with increased concentration in the utricle, dark cell region around the ampullae of the semicircular canals, and the endolymphatic duct (ED) (Kimura, 1969). Temporal bone studies have found that African–American individuals have increased pigment within the vestibular system (Erbele et al., 2016).

Few studies have quantitatively compared pigmentation within the auditory and vestibular systems of the same individuals or analyzed how pigmentation changes throughout adulthood in the mammalian inner ear. The goal of this study was to compare pigmentation within the auditory and vestibular system of mouse and human temporal bones.

## Materials and Methods

### Human Temporal Bone Subjects and Imaging

Human temporal bones were obtained from the Johns Hopkins Human Temporal Bone Collection (Nager Collection). These temporal bones were collected between the years of 1940 and 1988 and contain both pediatric and adult specimens. Demographic data consisting of age, sex, and race were available for all individuals. Specimens from adults that had available audiometric data were selected for analysis. The adult temporal bones studied were the same as those used in previous studies from our institution (Sun et al., 2014; Erbele et al., 2016). The pediatric temporal bones analyzed were from the same collection. Human temporal bone specimens were prepared using standard methods that have been described elsewhere (Crowe et al., 1934). In brief, fresh specimens were fixed in formalin, decalcified in nitric acid, dehydrated in alcohol baths, and then embedded in celloidin. The specimens were sectioned in 24 μM slices in the sagittal (vertical) plane and every 10th section was mounted on a glass slide and stained with hematoxylin and eosin (H&E).

Seventy-one human temporal bones were identified for analysis, of which 25 were from pediatric patients (mean age: 3.3 years; range 0–12 years) and 46 were from adult patients (mean age: 68 years; range 38–94 years). Thirteen of the pediatric temporal bones and 16 of the adult temporal bones were from females, respectively. Fourteen of the pediatric temporal bones were from Caucasian individuals and 11 were from African–American individuals. Twenty-seven of the adult temporal bones were from Caucasian individuals and 19 were from African–American individuals. The demographics of the human temporal bone cohort are described in Table 1.

**TABLE 1:** Description of demographic data for human temporal bone specimens.

**Age, years (range)**	
Pediatric	3.3 (0–12)
Adult	68 (38–94)
Gender, female (%)	
Pediatric	13 (52)
Adult	16 (35)
Race, *n* (%)	
Adult	46 (65)
Caucasian	27 (38)
African–American	19 (26)
Pediatric	25 (35)
Caucasian	14 (20)
African–American	11 (15)

**FIGURE 1:**
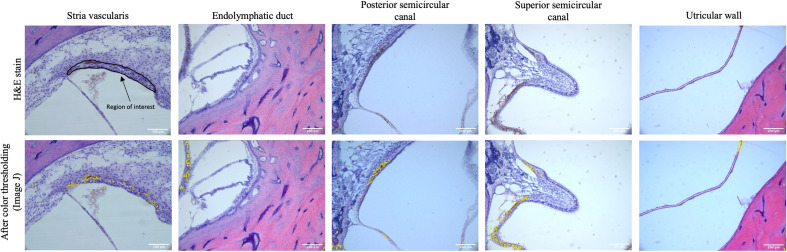
The top row shows representative images of the stria vascularis, endolymphatic duct, posterior semicircular canal, superior semicircular canal, and utricular wall in human temporal bones taken at 10× magnification using light microscopy. The stria vascularis is outlined (region of interest) in the top left panel. For the vestibular system the entire image as pictured above was used as the region of interest to measure pigment content. The bottom rows shows the images after color thresholding on ImageJ with the pigmented areas shaded in yellow.

Mid-modiolar images of the SV were then taken at 20× magnification using a digital camera mounted on a microscope with image acquisition software (Jenoptik ProgResCF). The vestibular system was imaged at 10× magnification at the following locations: the ED as it enters the vestibule prior to the endolymphatic sinus, the posterior semicircular canal (PSCC) as the posterior ampullary nerve enters, the ampulla of the superior semicircular canal (SSCC) with portions of the superior vestibular nerve, and the utricular wall (Figure 1).

### Mouse Temporal Bone Subjects and Imaging

CBA/CaJ mice were used to analyze age-related changes in pigmentation within the auditory and vestibular systems in a mouse strain that exhibits cochlear aging at a similar rate to humans when compared across the lifespan (Sergeyenko et al., 2013). Breeding pairs were originally obtained from the Jackson Laboratory and were bred in a quiet vivarium. Mice were housed individually or in groups with unrestricted access to food and water and kept on a 12:12 h day/night cycle. Some of these animals underwent auditory response brainstem (ABR) testing that has been described elsewhere (Kobrina et al., 2020). Temporal bones were harvested from mice at 3–4 weeks and 20–32 months of age. All procedures were approved by the Johns Hopkins University Animal Care and use Committee and complied with the ARRIVE guidelines and the associated NIH Guide for Care and Use of Laboratory Animals. In order to harvest temporal bones, animals were deeply anesthesized with a 0.3–0.5 mg/g intraperitoneal dose of pentobarbital, transcardially perfused with 60 mL of 4% paraformaldehyde fixative solution, and decapitated. The cochleas were reperfused with 4% paraformaldehyde through the oval and round windows, and then allowed to postfix overnight. Cochleas were then disarticulated from the skull and decalcified in 1% ethylenediaminetetraacetic acid (EDTA) for at least 1 week until spongy. Cochleas were dehydrated and embedded in Araldite and sectioned into 30 μM sections parallel to the modiolus with a rotary microtome following methods described by Schrode et al. (2018). The sections were mounted on subbed slides, stained with toluidine blue, and a coverslip was applied.

Nine juvenile (3–4 week) and 12 adult (20–32 months) CBA mice were identified for analysis. Six (66%) of the juvenile and eight (66%) of the aged mice were female. Five of the aged CBA mice were subjected to a 2 h 100 dB SPL octave band (8–16 kHz) noise exposure 3–4 months prior to tissue harvest. We included these specimens because recent work has suggested that much of the age-related degeneration reported in human temporal bone specimens is due to a history of noise exposure (Wu et al., 2020). Mouse models offer us the opportunity to look for potential additive effects of noise exposure on age-related inner ear pigmentation in a cohort of mice with otherwise similar life histories and genetic backgrounds.

Images of the SV were then taken at 20× magnification using a digital microscope mounted on a microscope with image acquisition software (Jenoptik ProgResCF). Images of the vestibular system were collected at 10× magnification in the same locations as for human temporal bones.

### Image Analysis

**FIGURE 2:**
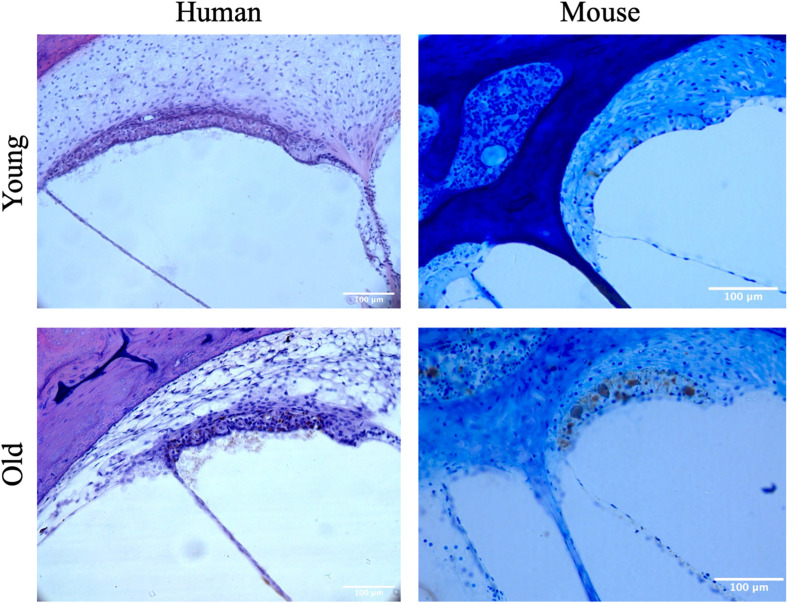
Representative images of the stria vascularis from young and aged mice and humans.

Images were analyzed using ImageJ (Bethesda, MD, United States). The cross-sectional area of the SV was determined by manually out-lining the border of the SV and using ImageJ. The area of the SV was measured in square micrometers after calibration with a scale bar. Pigmentation in the SV was measured using color thresholding (Figure 2), by a blinded observer, to measure the area (μM^2^) of SV containing pigment. The percentage of the SV containing pigment was then calculated for each image. The pigment content of the vestibular end organs was measured by a blind observer using color thresholding to select the area (μM^2^) containing pigment, which was then quantified as the area containing pigment per high power field (HPF).

### Statistics

Statistical analysis was performed using Stata 16 (College Station, Texas). Between group means were compared using a student’s *t*-test with a two-tailed *p*-value and a Bonferroni correction. The *p*-value for significance was set to 0.025 (0.05/2) when two comparisons were made (mouse vestibular system), 0.0125 (0.05/4) when four comparisons were made (mouse cochlea and human vestibular system), and 0.01 (0.01/5) when five comparisons were made (human cochlea). Within group tonotopic differences in SV pigmentation were tested with a one-way ANOVA test. Multivariable liner regression was performed to assess the effect of gender, age, and race on the pigment content with the SV of human temporal bones. For the human temporal bones, a comparison of the pigment content of the SV and audiometric thresholds has been performed previously and described elsewhere (Sun et al., 2014).

## Results

### Age-Related Effects on Pigmentation Within the SV

**FIGURE 3:**
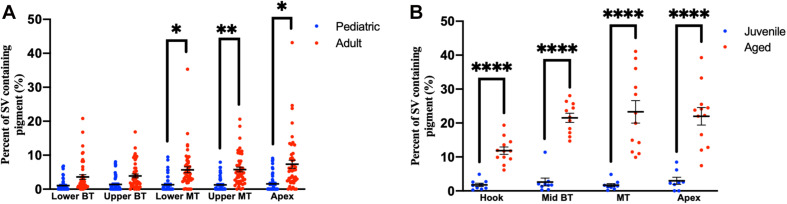
Percentage of the stria vascularis containing pigment within human **(A)** and mouse **(B)** temporal bones. Error bars indicate standard error. Stria vascularis, SV; BT, basal turn; MT, middle turn. ^∗^Adjusted *p*-value < 0.05, ^∗∗^adjusted *p*-value < 0.01, and ^*⁣*⁣**^adjusted *p*-value < 0.0001.

**FIGURE 4:**
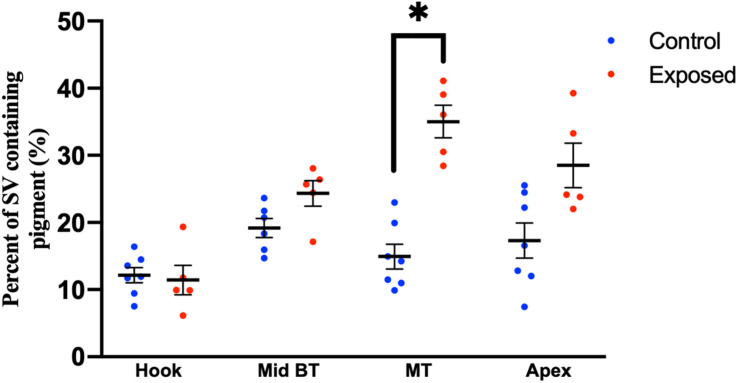
Percentage of the stria vascularis containing pigment within adult (20–32 month) mouse temporal bone specimens with or without noise exposure. Error bars indicate standard error. ^∗^Adjusted *p*-value < 0.05.

Compared to pediatric human temporal bones, the adult temporal bones had increased pigmentation within the SV of the lower middle turn (*p* = 0.008, *t* = 2.745, 95% CI = 1.06–6.32%), upper middle turn (*p* = 0.0013, *t* = 3.36, 95% CI = 1.54–5.84%), and apex (*p* = 0.012, *t* = 2.57, 95% CI = 1.11–8.23%). The comparison of SV pigmentation in pediatric and adult human temporal bones is shown in Figure 3A. Among the mouse temporal bone specimens, there was increased pigmentation within the SV of aged mice, compared to juvenile mice, at the hook (*p* < 0.00001, *t* = 7.66, 95% CI = 7.39–12.67%), mid-basal turn (*p* < 0.00001, *t* = 10.50, 95% CI = 15.31–22.51%), middle turn (*p* < 0.00001, *t* = 9.45, 95% CI = 17.05–26.21%), and apex (*p* < 0.00001, *t* = 6.10, 95% CI = 12.77–25.21%). The results for SV pigmentation in juvenile and aged mice are shown in Figure 3B. Aged mice subjected to noise exposure had increased pigment within the SV of the middle turn (*p* = 0.00005, *t* = 7.74, 95% CI = 17.14–23.12%), but not the hook (*p* = 0.89, *t* = 0.14, 95% CI = −4.21 to 4.84%), mid-basal turn (*p* = 0.04, *t* = 2.35, 95% CI = 0.76–9.64%), or apex (*p* = 0.02, *t* = 11.20, 95% CI = 2.83–19.57%) compared to aged mice without noise exposure. The results for noise exposure are shown in Figure 4. One-way ANOVA test showed tonotopic differences in SV pigmentation in adult human (*p* = 0.005; *F* = 3.82) and aged mouse (*p* = 0.0002; *F* = 8.37) temporal bones, but not pediatric human (*p* = 0.81; *F* = 0.39) or juvenile mouse (*p* = 0.62; *F* = 0.60) temporal bones.

### Age-Related Effects on Pigmentation Within the Vestibular System

**FIGURE 5:**
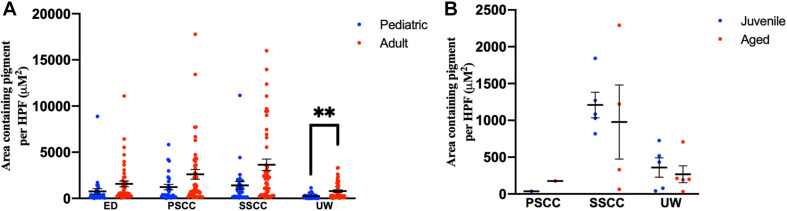
Pigmentation within the vestibular system in human **(A)** and mouse **(B)** temporal bones. Error bars indicate standard error. ED, endolymphatic duct; PSCC, posterior semicircular canal; SCCC, superior semicircular canal; UW, utricle wall. ^∗∗^Adjusted *p*-value < 0.01.

Adult human temporal bones had increased pigmentation within the utricular wall (*p* = 0.008, *t* = 2.73, 95% CI = 145–940 μM^2^/HPF), but not the ED (*p* = 0.12, *t* = 1.56, 95% CI = −229 to 1,851 μM^2^/HPF), superior semicircular canal (SSCC) (*p* = 0.018, *t* = 2.42, 95% CI = 386–4,052 μM^2^/HPF), or PSCC (*p* = 0.09, *t* = 1.71, 95% CI = −233 to 2,987 μM^2^/HPF), compared to pediatric temporal bones. The results for the pigmentation of the human vestibular system are shown in Figure 5A. Aged mice did not have increased pigmentation of the superior semicircular canal (SSCC) (*p* = 0.65, *t* = 0.48, 95% CI = −737 to 1,199 μM^2^/HPF) or utricular wall (UW) (*p* = 0.23, *t* = 1.30, 95% CI = 146–686 μM^2^/HPF). The results for the analysis of the mouse vestibular system are shown in Figure 5B.

### Ethnicity and Inner Ear Pigmentation

**FIGURE 6:**
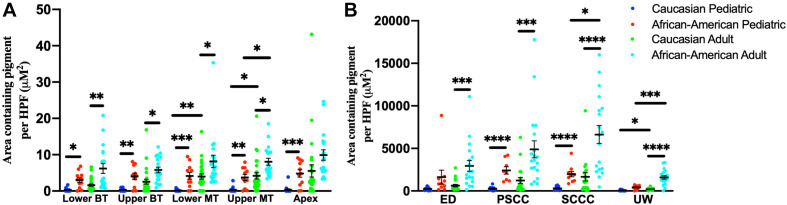
Pigmentation within the stria vascularis **(A)** and vestibular system **(B)** of human temporal bones, broken down by age and race. Error bars indicate standard error. SV, stria vascularis, BT, basal turn; MT, middle turn; ED, endolymphatic duct; PSCC, posterior semicircular canal; SCCC, superior semicircular canal; UW, utricle wall. ^∗^Adjusted *p*-value < 0.05, ^∗∗^adjusted *p*-value < 0.01, ^∗∗∗^adjusted *p*-value < 0.001, and ^*⁣*⁣**^adjusted *p*-value < 0.0001.

Among adult human temporal bone specimens from African–American individuals, there was increased pigmentation of the SV at the lower basal turn (*p* = 0.0004, *t* = 3.84, 95% CI = 2.19–6.96%), upper basal turn (*p* = 0.003, *t* = 3.14, 95% CI = 1.18–5.32%), lower middle turn (*p* = 0.016, *t* = 2.50, 95% CI = 0.84–7.37%), and upper middle turn (*p* = 0.005, *t* = 2.97, 95% CI = 1.32–6.46%) compared to Caucasian adults. Among pediatric human temporal bone specimens from African–American individuals, there was increased pigmentation of the SV at the lower basal turn (*p* = 0.005, *t* = 3.10, 95% CI = 0.84–3.72%), upper basal turn (*p* = 0.0008, *t* = 3.83, 95% CI = 1.59–4.95%), lower middle turn (*p* = 0.0003, *t* = 4.22, 95% CI = 2.00–5.45%), upper middle turn (*p* = 0.0005, *t* = 4.04, 95% CI = 1.59–4.61%) and apex (*p* = 0.0001, *t* = 4.57, 95% CI = 2.43–6.07%) compared to Caucasian individuals. African–American adult specimens had increased pigmentation within the SV of the upper middle turn (*p* = 0.004, *t* = 3.12, 95% CI = 1.63–7.07%), but not the lower basal turn (*p* = 0.10, *t* = 1.72, 95% CI = −0.45–6.84%), upper basal turn (*p* = 0.15, *t* = 1.47, 95% CI = −0.58 to 4.16%), lower middle turn (*p* = 0.10, *t* = 1.69, 95% CI = −0.65 to 8.61), or apex (*p* = 0.019, *t* = 2.49, 95% CI = 1.07–9.11%) compared to African–American pediatric specimens. Caucasian adult specimens had increased pigmentation within the SV of the lower middle turn (*p* = 0.0006, *t* = 3.72, 95% CI = 1.69–5.45%) and upper middle turn (*p* = 0.004, *t* = 3.06, 95% CI = 1.29–5.83%), but not the lower basal turn (*p* = 0.09, *t* = 1.71, 95% CI = −0.12 to 1.92%), upper basal turn (*p* = 0.06, *t* = 1.94, 95% CI = −0.01 to 3.63%), or apex (*p* = 0.029, *t* = 2.26, 95% CI = 0.66–9.28%) compared to Caucasian pediatric specimens. The results for pigmentation of the SV by age and race are shown in Figure 6A.

For temporal bone specimens from African–American adults there was increased pigmentation of the endolymphatic duct (ED) (*p* = 0.00009, *t* = 4.29, 95% CI = 1,257–3,369 μM^2^/HPF), PSCC (*p* = 0.00008, *t* = 4.34, 95% CI = 2,122 to 5,622 μM^2^/HPF), superior semicircular canal (SSCC) (*p* < 0.00001, *t* = 5.27, 95% CI = 3,255–7,109 μM^2^/HPF), and utricular wall (UW) (*p* < 0.00001, *t* = 7.42, 95% CI = 979–1,681 μM^2^/HPF) compared to Caucasian adults. Among pediatric temporal bones, there was increased pigmentation within the PSCC (*p* < 0.00001, *t* = 6.38, 95% CI = 1,482–2,798 μM^2^/HPF), superior semicircular canal (SSCC) (*p* < 0.00001, *t* = 6.03, 95% CI = 1,130–2,220 μM^2^/HPF) and utricular wall (UW) (*p* = 0.00004, *t* = 5.04, 95% CI = 209–475 μM^2^/HPF) of African–American individuals compared to Caucasian individuals. African–American adult specimens had increased pigmentation within the superior semicircular canal (SSCC) (*p* = 0.0026, *t* = 3.30, 95% CI = 1,897–7,428 μM^2^/HPF) and utricular wall (UW) (*p* = 0.0002, *t* = 4.30, 95% CI = 643–1,721 μM^2^/HPF), but not the endolymphatic duct (ED) (*p* = 0.20, *t* = 1.30, 95% CI = −663 to 2,332 μM^2^/HPF) or PSCC (*p* = 0.08, *t* = 1.84, 95% CI = −168 to 5,132 μM^2^/HPF) compared to African–American pediatric specimens. Caucasian adult specimens had increased pigmentation within the utricular wall (UW) (*p* = 0.006, *t* = 2.87, 95% CI = 62–326 μM^2^/HPF), but not the endolymphatic duct (ED) (*p* = 0.017, *t* = 2.49, 95% CI = 94–780 μM^2^/HPF), PSCC (*p* = 0.05, *t* = 1.99, 95% CI = 14–1,487 μM^2^/HPF), or superior semicircular canal (SSCC) (*p* = 0.03, *t* = 2.87, 95% CI = 140–2,170-μM^2^/HPF) compared to Caucasian pediatric specimens. Measurements for pigmentation of the vestibular system by age and race are shown in Figure 6B.

Multivariable linear regression analysis for the total pigment content within the SV of human temporal bones showed that increased age (*p* < 0.001, *t* = 4.93, 95% CI = 0.03–0.07%) and African–American race (*p* < 0.001, *t* = 6.06, 95% CI = 2.78–5.51%) were associated with increased pigmentation, but pigmentation was not influenced by gender (*p* = 0.28, *t* = 1.10, 95% CI = −0.6 to 2.13%).

## Discussion

The present study found that pigmentation increases with age in the SV of the mammalian inner ear, but does not clearly increase with age in the vestibular system. Specifically, pigmentation within the SV of human temporal bones increased from childhood to adulthood, and these changes correlated with similar findings in a mouse model of age-related hearing loss. Amongst the mouse temporal bones noise exposure resulted in increased pigmentation with the SV of the middle turn, but not other regions of the cochlea. It was not possible to control for noise exposure amongst the human temporal bones and it is possible that some of the increased pigmentation seen with age is related to noise exposure. Pigmentation within the vestibular system increased with age in the utricular wall of human temporal bones, but this relationship was unclear in other regions of the vestibular system and did not hold true in mouse temporal bones. Skin color was also found to correlate with inner ear pigmentation in human temporal bones, both in childhood and adulthood. This association between skin color and inner ear pigmentation has previously been described (Sun et al., 2014; Erbele et al., 2016), but this study is the first to describe differences in inner ear pigmentation during childhood and the increase in pigmentation observed with age.

Pigment has long been proposed to play an important role in the function of the inner ear. In 1965 Bonaccorsi hypothesized that eye color may predict inner ear pigmentation and provide protection against noise-induced hearing loss (Bonaccorsi, 1965). However, current evidence suggests that the relationship between eye color and cochlear pigmentation is unclear (Mujica-Mota et al., 2015). Inner ear pigmentation has been hypothesized to protect against oxidative injury and maintaining calcium homeostasis by acting as a metal ion chelator (Bush and Simon, 2007; Laurell et al., 2007; Murillo-Cuesta et al., 2010; Fetoni et al., 2019). Cochlear pigmentation appears to be protective against radiation-induced sensorineural hearing loss (Mujica-Mota et al., 2015). Previous studies have reported cochlear pigmentation to be increased in African–American individuals (Sun et al., 2014) and decreased rates of hearing loss in African–American individuals on population based studies. The similar changes observed within the SV in both humans and mouse model highlight the potentially important role of pigmentation within the inner ear. Pigmentary defects are known to cause hearing loss in both humans (Lezirovitz et al., 2006; Léger et al., 2012) and animal models (Keithley et al., 1992; Ohlemiller et al., 2009), and the similar changes observed within the SV across mammalian models highlights that pigment may play a fundamental and important role in the cochlea.

Whether pigment plays a protective role against noise- or age-related hearing loss remains unclear. While individuals with darker skin have a lower incidence of hearing loss (Ishii and Talbott, 1998; Lin et al., 2012) the actual function of melanin within the SV has yet to be elucidated. Increased pigmentation in the SV from African–American pediatric temporal bones suggests that melanin is present at a young age and thus could be present to play a protective role against age-related degeneration or accrual of noise-induced hearing loss over the course of a lifetime. However, lipofuscin is also known to increase with age, and some of the increased pigmentation observed, particularly within the mouse model, may be the result of lipofuscin accumulation. The fact that pigment increases with age and is often concurrent with presbycusis raises the possibility that pigmentation is simply a metabolic by-product that may not serve a protective role.

The observed age-related changes in pigmentation within the SV highlights the need for precise identification of the site of otologic pathology in age-related hearing-loss, which has been a subject of controversy and conflicting reports. Early temporal bone studies identified neural degeneration and outer hair cell (OHC) loss as principal components of presbycusis (Guild et al., 1931; Crowe et al., 1934). A primary role for OHC loss in presbycusis is supported by a more recent human temporal bone study that used statistical modeling and immunolabeling to more precisely measure neural degeneration (Wu et al., 2020). However, other human temporal bone studies have pointed to SV degeneration as a principal mechanism of age-related hearing loss (Schuknecht et al., 1974), and numerous animal studies have implicated the SV in age-related hearing loss (Strain, 2004, 2017; Ohlemiller et al., 2006, 2009). Part of this discrepancy may be explained by the fact that human temporal bone studies are limited to histopathologic correlations and can only measure atrophy of the SV, rather than function. However, further study is warranted given the importance of this question in the development of targeted therapies for presbycusis.

Pigmentary changes within the vestibular system are comparatively less understood. It has been over 50 years since Kimura first provided a detailed description of the distribution of dark cells and subepithelial melanocytes within the vestibular system of animal models and humans (Kimura, 1969). However, little is understood regarding the function of pigment within the vestibular system. Much of this knowledge deficit is likely related to the difficulty of studying vestibular function relative to hearing. Our findings build upon the findings of Erbele et al. (2016) by showing that race was associated with increased pigmentation of the vestibular system in childhood, as well as adulthood, and these pigmentary changes increase with age. There is some evidence that individuals with darker skin may have improved vestibular function (Agrawal et al., 2008; Li et al., 2015), but this association has been questioned when less sensitive measures of vestibular function were used (Agrawal et al., 2009). In the mouse temporal bone specimens we were unable to demonstrate a difference in pigmentation with age, which may have been the result of inadequate sample size or could mean that there is in fact no difference with age. Previous studies have shown that humans and mice have a similar distribution of pigment-containing cells within the vestibular system (Kimura, 1969; LaFerriere et al., 1974). The observed differences in age-related pigmentary changes in the cochlea and vestibular system may be related to the different origins of cochlear and vestibular melanocytes (Bonnamour et al., 2020) or that the lifelong presence of dark cells accounts for a large amount of vestibular pigmentation (Kimura, 1969). Changes in vestibular pigmentation with age, skin pigmentation, or inner-ear insults such as noise exposure are poorly understood. In particular, no data exists describing changes in pigmentation in relation to vestibular function.

This study has several limitations. First, we are limited to the use of race as a surrogate for Fitzpatrick skin type, which is both an indirect and possibly imprecise measure of skin pigmentation. There may also be differences in SV pigmentation in mice related to coat color, which was not addressed in this study due to the use of a single mouse strain. Future studies should further examine strain- and coat-related differences in SV pigmentation (Bartels et al., 2001). Further, the techniques used in this study are not capable of unambiguously differentiating between different types of pigment. Lipofuscin is known to accumulate in cells with age and may have been inadvertently measured as pigment. There are also different types of melanin, eumelanin and pheomelanin, that we were unable to differentiate among in this study. In addition to this, the cross-sectional nature of this study limits the inferences that can be made regarding the actual function of melanin in the inner ear. Future studies are needed that specifically analyze the biologic role of melanin in the aging auditory and vestibular periphery. Comparison of albino and darkly pigmented mice may provide further insight into the role of pigment in inner ear function (Murillo-Cuesta et al., 2010; Gi et al., 2018). The development of *in vivo* pharmacologic techniques to manipulate melanin concentration within the ear may allow us to better understand the role of pigment within the inner ear.

In summary, this study showed that pigmentation within the cochlea increases with age in mouse and human temporal bone specimens. Pigmentation within the vestibular system increased with age in the utricular wall of human temporal bones, but did not increase in other human vestibular end organs and did not increase with age in the mouse vestibular system. Individuals who identified as African–American had higher pigment content within the SV and vestibular epithelia, both as juveniles and as adults. These results highlight how similar age-related pigmentary changes occur in the auditory systems across species. Our findings also underscore the need for representation of participants who reflect the racial/ethnic diversity of the aging population both within temporal bone studies and within studies of inner ear function more broadly.

## Data Availability Statement

The raw data supporting the conclusions of this article will be made available by the authors, without undue reservation.

## Ethics Statement

Ethical review and approval was not required for the study on human participants in accordance with the local legislation and institutional requirements. Written informed consent for participation was not required for this study in accordance with the national legislation and the institutional requirements. The animal study was reviewed and approved by the Johns Hopkins University Animal Use and Care Committee.

## Author Contributions

NA and AL conceptualized the project and drafted the initial manuscript. NA, SC, and DV performed the data collection and analysis. NA, SC, DV, and AL interpreted the data. All authors contributed to the writing.

## Conflict of Interest

The authors declare that the research was conducted in the absence of any commercial or financial relationships that could be construed as a potential conflict of interest.
